# Airway Proficiency and Efficiency Amongst Anesthesia Providers and Respiratory Therapists: A Comparison Study

**DOI:** 10.3390/jcm14228059

**Published:** 2025-11-13

**Authors:** Calleigh G. Brignull, Emily B. Williams, Harper A. Sprouse, Kyle J. Adams, Stephanie L. Tanner, John W. Sykes, Henry Moulder, William R. Hand, Robert R. Morgan

**Affiliations:** 1 Department of Anesthesiology, Prisma Health-Upstate, Greenville, SC 29605, USAkyle.adams@prismahealth.org (K.J.A.); stephanie.tanner@prismahealth.org (S.L.T.); william.hand@prismahealth.org (W.R.H.);; 2 Prisma Health Upstate Simulation Center, Greenville, SC 29605, USA; henry.moulder@prismahealth.org

**Keywords:** airway management, direct laryngoscopy, video laryngoscopy, laryngeal mask airway, clinical skills

## Abstract

**Background/Objectives**: Studies have demonstrated significant morbidity and mortality associated with airway management, especially when provided outside of the operative setting. The goal of this study was to compare baseline measurements of airway management procedures between anesthesia providers (CRNAs and anesthesiologists) and respiratory therapists using high-fidelity manikins. **Methods**: This prospective study assessed anesthesia providers and respiratory therapists performing direct laryngoscopy (DL), video laryngoscopy (VL), and LMA placement. The same Laerdal SimMan high-fidelity manikin (Laerdal, Stavanger, Norway) was used in all assessments, with the detection of end-tidal “carbon dioxide” serving as evidence of success for each procedure. Each procedure was performed twice, once under “Healthy Patient” SimMan settings, and once under the “Limited Cervical Range of Motion (ROM)” (DL), “Pharyngeal Obstruction” (VL), and “Full Tongue Edema” (LMA) settings, respectively, to simulate a moderately difficult airway. The order in which the techniques were performed was randomized for each participant. Completion time and number of attempts were recorded for each procedure and compared between the groups. **Results**: Sixty-two providers (30 anesthesia providers and 32 respiratory therapists) were enrolled. There were no significant differences in average time to completion for any procedure, except respiratory therapists took longer than anesthesia providers in VL with simulated pharyngeal obstruction (*p* = 0.0004). There were no differences in number of attempts needed for successful completion. **Conclusions**: This study demonstrates that while completion times for DL and LMA placement were similar amongst provider groups, average time to completion of VL for respiratory therapists was longer under difficult simulated settings. These results reflect potential areas of improvement for other provider groups that may have airway privileges at their respective institutions.

## 1. Introduction

Airway management skills require familiarity and competency with a wide variety of procedural techniques and devices. Skill acquisition typically takes place during healthcare providers’ respective training programs, with proficiency largely maintained by “on the job” experience. Previous studies have demonstrated that the time required to perform many of the techniques required for airway management may vary with experience and the type of provider [1].

Prior research has also demonstrated significant morbidity and mortality associated with airway management, especially when such care is provided outside the operating room environment [2,3,4,5,6,7,8]. Brindley et al. found that hypotension is frequently associated with the induction of anesthesia for airway management purposes, and Cook et al. noted a 38-fold higher frequency of airway complications in the emergency department and a 58-fold higher frequency in the Intensive Care Unit as compared to the same procedures being performed in the operating room setting.

Many hospitals and health systems assign airway management privileges to a diverse array of clinical providers, including (but not limited to) advanced practice registered nurses, physician assistants, respiratory therapists, certified registered nurse anesthetists (CRNAs), anesthesiology assistants (AAs), and multiple physician specialists [9]. These provider groups may have widely disparate experiences with airway management given their training, ongoing clinical experience, and the policies and procedures unique to any hospital or health system’s clinical environment [10]. 

The circumstances surrounding airway management may also vary widely, ranging from an elective surgical procedure in which providers typically have ample time to gather supplies and formulate a plan, to medical emergencies in which airway management must be performed as rapidly as possible. To provide a logical framework for approaching airway management, the American Society of Anesthesiologists first developed the Difficult Airway Algorithm (DAA) in 1993 [11,12].

While other professional organizations have similarly created or adapted the DAA for their purposes, most of these share common characteristics and presume a basic familiarity with several airway devices, ranging from supraglottic approaches (ex: direct or video laryngoscopy) to subglottic techniques (ex: cricothyrotomy) [13]. Knowledge of those algorithms, as well as proficiency with the psychomotor skills needed to perform those techniques, is fundamental to success and quality outcomes for patients. Failure to adhere to these algorithms can lead to adverse outcomes, ranging from minor injury to death [14,15].

To assess the airway management skills of the diverse array of clinicians in a healthcare system, it is necessary to establish a baseline level of competency to compare the skills of various provider groups. Given the training and ongoing clinical experience of both anesthesiologists and CRNAs, we proposed quantitatively measuring their success rates and times of completion as benchmarks with which other providers could be compared. In most sizable anesthesia departments across the country, these providers completed training at a wide variety of programs, thus providing geographic and institutional diversity as it relates to their background experience. To date, there have been few other studies dedicated to the baseline assessment of such skills using high-fidelity simulators to capture success rates and completion times.

The overall goal of this study was to compare baseline measurements of various airway management procedures amongst anesthesia providers, who are generally acknowledged to be airway experts, and respiratory therapists, who frequently perform the same procedures, using high-fidelity manikins. The hypothesis was that anesthesia providers would have shorter completion times and less required attempts than respiratory therapists for each of the three airway management procedures and both difficulty settings.

## 2. Materials and Methods

### 2.1. Study Population

This study included anesthesia providers and respiratory therapists from the same department(s) at a single institution. Local Institutional Review Board approval was obtained prior to enrollment and written informed consent was obtained from all eligible participants. All anesthesia providers and respiratory therapists with active airway management privileges at a single institution were offered participation in this study from October 2021–March 2025. Participants were excluded from the study if they declined to provide informed consent. At the time of consent, each participant’s provider type and self-reported years of airway management experience were recorded.

### 2.2. Study Design

**Figure 1:**
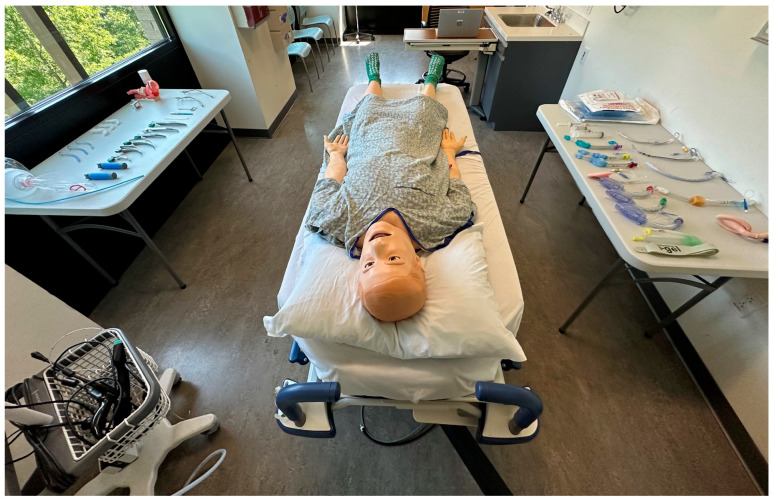
View from the manikin head depicting room setup and available equipment.

**Figure 2:**
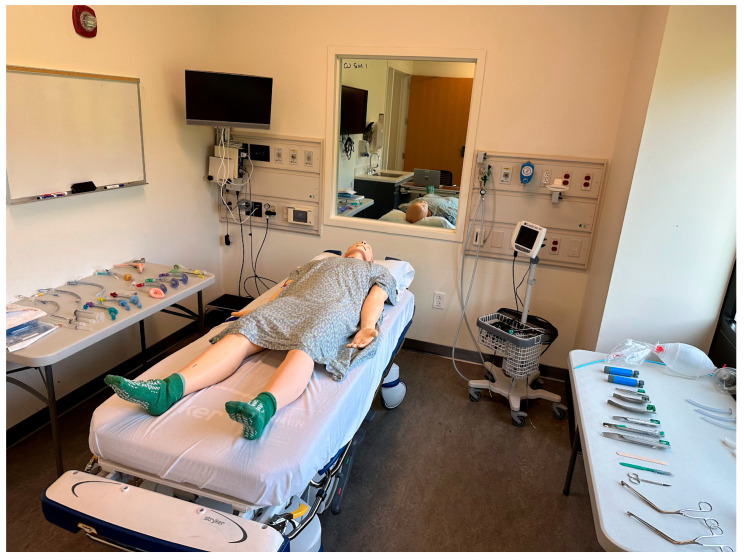
Overview of manikin, room layout, and available equipment.

This was a prospective study assessing the proficiency and efficiency of airway management techniques amongst anesthesia providers and respiratory therapists. The three procedures performed were direct laryngoscopy (DL), video laryngoscopy (VL) utilizing the Verathon Glidescope Video Monitor (Verathon, Bothel, WA, USA), and laryngeal mask airway (LMA) placement. The same Laerdal SimMan high-fidelity manikin (Laerdal, Stavanger, Norway) was used in all assessments, with the detection of end-tidal “carbon dioxide” serving as evidence of success for each procedure. Each procedure was performed twice, once under “Healthy Patient” SimMan settings, and once under the “Limited Cervical Range of Motion (ROM)” (DL), “Pharyngeal Obstruction” (VL), and “Full Tongue Edema” (LMA) settings, respectively, to simulate a moderately difficult airway. The order in which the techniques were performed was randomized for each participant and each participant was blinded to the participants. During testing of each airway management procedure, the participants had the opportunity to adjust the bed height, gather the supplies needed, and position the manikin’s head prior to initiating the procedure (Figure 1 and Figure 2).

**Figure 3:**
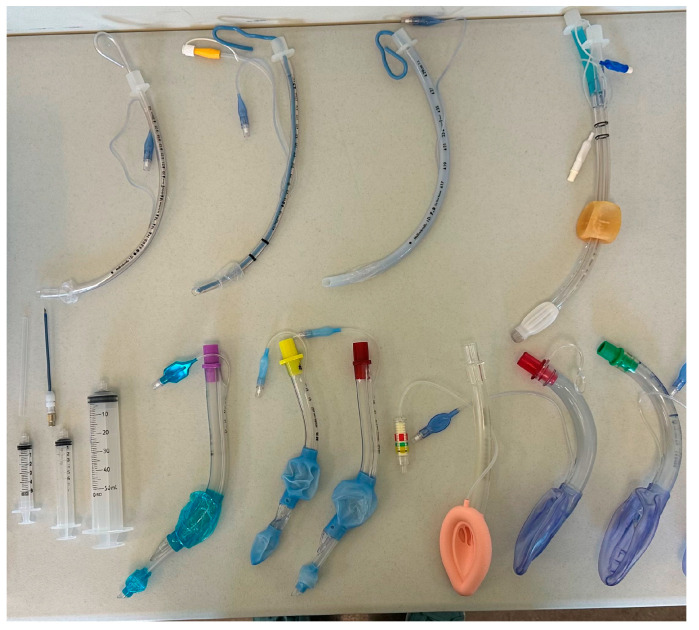
Layout of the equipment available to each participant performing the simulation, table one of two.

A standard laryngoscope handle and Macintosh (sizes 3 and 4) and Miller blades (sizes 2 and 3) were offered to each participant performing DL. The participants had the opportunity to choose any of the four laryngoscope blades for DL and could change blades during the performance of the procedure as desired. A 7.0 Mallinckrodt endotracheal tube (Mallinckrodt, St. Louis, MO, USA) was provided for all DL attempts (Figure 3).

**Figure 4:**
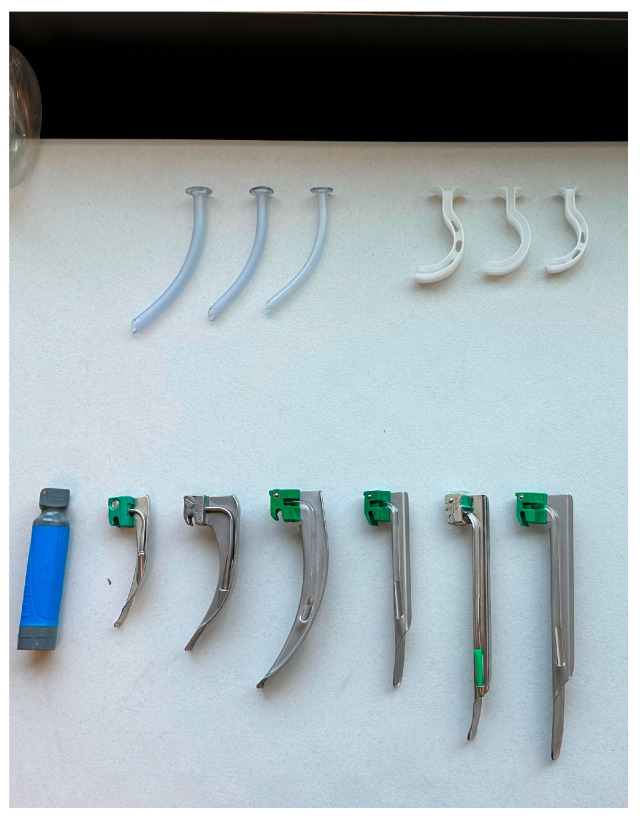
Layout of the equipment available to each participant performing the simulation, table two of two.

A video laryngoscope with a non-disposable handle and sizes 3 and 4 blades were offered to each participant performing VL (Figure 4). The participant had the opportunity to choose either of the two laryngoscope blades for VL and could change blades during the performance of the procedure as desired. A 7.0 Mallinckrodt endotracheal tube was provided for all VL attempts. A traditional endotracheal tube stylet was available as well as a rigid stylet that is typically provided for use with most VL systems. The participant chose either stylet as desired.

Three Air-Q LMAs (sizes 3, 4, and 5) were offered to each participant performing LMA placement. The participant had the opportunity to choose any of the LMAs and could change LMAs during the performance of the procedure as desired.

Time measurement for all three procedures began when the participant picked up the laryngoscope, video laryngoscope, or LMA, having signaled his/her intention to begin the procedure. Time measurement ended upon detection of end-tidal “carbon dioxide” by the manikin, as evidenced on a standard patient monitor connected to the manikin for this purpose. Failed attempts for any of the three procedures were documented when a participant attached a bag/valve device and attempted ventilation for purposes of confirming the presence of end-tidal “carbon dioxide” (which was not displayed if the device was improperly placed) or the participant changed the device and repositioned the manikin. Time measurement continued during failed attempts and subsequent efforts to successfully perform the procedure. Time measurement was stopped upon successful end-tidal “carbon dioxide” measurement, three sequential failed attempts to perform a procedure, or after five minutes had passed.

### 2.3. Statistical Analysis

Randomization of the procedures for each participant was computer-generated through REDCap [16,17]. Randomization occurred after informed consent was obtained. The participants performed each airway technique based on the order that the technique was randomized.

All study data were recorded on customized case report forms and then entered in REDCap for data storage [16,17]. No a priori power analysis was performed. Standard descriptive statistics were used to assess provider demographics, procedural success rates, and the duration of each procedure for all providers.

The normality assumptions for the time to successful completion for each airway management procedure and difficulty setting were first checked using Shapiro–Wilk tests and visually with QQ plots. The normality assumptions were not met, thus the time to successful completion between the two provider types for each airway management procedure were analyzed using Mann–Whitney U tests. The number of attempts required between the two provider types for each procedure were analyzed using Fisher’s Exact Tests. The significance level for all statistical analyses was set as a priori to alpha = 0.05 and no statistical adjustments were made for multiple comparisons. All statistical analyses were performed using R Version 4.4.3.

## 3. Results

A total of 62 providers met the inclusion criteria and participated in the study, 30 of whom were anesthesiologists or CRNAs and 32 of whom were respiratory therapists. No providers were excluded. The median length of experience for the anesthesia providers was 9 years (range 8 months–36 years) and for respiratory therapists was 14.5 years (range 6 months–50 years).

The median time to completion (in seconds) for each of the airway procedures is shown in Table 1. There were no statistically significant differences in average time to completion for any of the procedures, except respiratory therapists took significantly longer than anesthesia providers in the VL with simulated pharyngeal obstruction setting (*p* = 0.0004).

The number of attempts needed for successful completion of each airway procedure is shown in Table 2. There were no significant differences between the anesthesia providers and respiratory therapists across the three procedures for easy or more difficult simulated settings. A total of 21.9% of respiratory therapists required two or more attempts compared to 3.3% of anesthesia providers for VL when faced with pharyngeal obstruction, although that number did not reach statistical significance (*p* = 0.0540).

## 4. Discussion

Establishing baseline levels of competency for various airway management procedures can provide benchmarks with which different provider groups may be compared. Furthermore, directly assessing performance with two levels of simulated airway difficulty may help to identify skill variations amongst these groups. Such variability could indicate the need for additional or ongoing training to minimize the risks to patients undergoing airway management procedures.

The authors are not aware of any standardized requirements for airway management training in the various programs from which healthcare providers may emerge to ultimately be granted privileges to perform such procedures. In some cases, there may be recommendations or guidelines, such as a minimum number of endotracheal intubation attempts, for any given training program, but there is little or no evidence that such guidance produces competency in completing those procedures for graduates of those programs [18,19].

Furthermore, the experience of healthcare providers may vary tremendously once they are in clinical practice when it comes to airway management procedures. Some groups, such as respiratory therapists, may perform such procedures on a regular and ongoing basis, while others may be called upon to do so only a limited number of times any given year. Given the potential for significant morbidity and mortality associated with such procedures, we believe that patient safety concerns compel us to establish baseline numbers that can be compared across various provider groups [6,15]. 

The findings of this study suggest that there may indeed be variations across such provider groups, even amongst two cohorts (anesthesia provider and respiratory therapists) who typically have the most experience with airway management. While “easy” or “routine” airways may be relatively easy to manage, when providers encounter more challenging circumstances such as pharyngeal obstruction in this simulation study, the time required to successfully perform airway procedures could lengthen significantly. In light of findings by Martin et al. indicating a difficult intubation rate of 10.3% outside of the operating room environment, and given the aforementioned morbidity and mortality associated with such efforts, the additional time required and/or increased number of attempts may have a direct impact on patient outcomes [2]. While factors such as pre-oxygenation may provide additional time for completion of such procedures, in many cases the provision of supplemental and/or pre-procedural oxygen may not be possible. In short, time is of the essence.

Limitations of this study include relatively small sample sizes for both anesthesia providers and respiratory therapists. No statistical adjustments were made for multiple comparisons, which can increase the Type I error rate. While time in clinical practice may not directly correlate to the number of nor frequency in performing the airway procedures included in this study, the authors acknowledge that experience may vary both within the two groups assessed and across those groups as well. It was assumed that both groups routinely performed the three airway procedures selected for this study, although participants were not asked for an estimate of how many such procedures they had performed in the recent past. Additionally, performance on a manikin could differ from performance on actual patients in a clinical setting.

## 5. Conclusions

These findings suggest that respiratory therapists may perform comparably in terms of the attempts needed and time taken to anesthesia providers with DL and LMA placement under both easy and more difficult simulated airway settings. The performance of respiratory therapists was also comparable using video laryngoscopy under easy settings but took significantly longer under more difficult settings.

Future research with larger sample sizes should be conducted to further assess and validate these findings. Additional provider groups should also be evaluated for similar variations given the significant morbidity and mortality associated with airway management. Providers and health systems might utilize such findings to design ongoing training programs to ensure uniformity in skill levels across all provider groups with privileges in airway management procedures.

## Figures and Tables

**Table 1:** Comparison of Median Time to Completion for Anesthesia Providers vs. Respiratory Therapists.

Task	Anesthesia Providers (*n* = 30) Median Time to Complete (Range) (Seconds)	Respiratory Therapists (*n* = 32) Median Time to Complete (Range) (Seconds)	*p*-Value	95% CI
Direct Laryngoscopy, Healthy Patient	21.4 (12.0–86.1)	23.2 (13.6–90.4)	0.1999	[−6.12, 1.33]
Video Laryngoscopy, Healthy Patient	23.0 (13.4–109.0)	26.0 (14.4–218.0)	0.2598	[−6.68, 2.16]
LMA Placement, Healthy Patient	14.2 (5.6–111.0)	17.0 (8.8–99.2)	0.0807	[−6.47, 0.48]
Direct Laryngoscopy, Limited Cervical ROM	30.9 (16.3–165.0)	31.1 (17.0–139.0)	0.8512	[−6.51, 9.47]
Video Laryngoscopy, Pharyngeal Obstruction	23.7 (9.3–74.2)	43.3 (17.8–120.0)	0.0004	[−27.30, −5.90]
LMA Placement, Full Tongue Edema	15.4 (6.3–47.5)	16.1 (8.8–274.0)	0.2628	[−5.97, 1.91]

**Table 2:** Comparison of Number of Attempts Required for Anesthesia Providers vs. Respiratory Therapists.

Task	Anesthesia Providers (*n* = 30) Number of Attempts Required (%)	Respiratory Therapists (*n* = 32) Number of Attempts Required (%)	*p*-Value	95% CI
Direct Laryngoscopy, Healthy Patient	1 attempt = 29 (96.7%) 2+ attempts = 1 (3.3%)	1 attempt = 31 (96.9%) 2+ attempts = 1 (3.1%)	1	[0.01, 75.89]
Video Laryngoscopy, Healthy Patient	1 attempt = 28 (93.3%) 2+ attempts = 2 (6.7%)	1 attempt = 28 (87.5%) 2+ attempts = 4 (12.5%)	0.6724	[0.26, 23.54]
LMA Placement, Healthy Patient	1 attempt = 28 (93.3%) 2+ attempts = 2 (6.7%)	1 attempt = 30 (93.8%) 2+ attempts = 2 (6.2%)	1	[0.06, 13.70]
Direct Laryngoscopy, Limited Cervical ROM	1 attempt = 28 (93.3%) 2+ attempts = 2 (6.7%)	1 attempt = 27 (84.4%) 2+ attempts = 5 (15.6%)	0.4267	[0.38, 29.00]
Video Laryngoscopy, Pharyngeal Obstruction	1 attempt = 29 (96.7%) 2+ attempts = 1 (3.3%)	1 attempt = 25 (78.1%) 2+ attempts = 7 (21.9%)	0.0540	[0.91, 377.55]
LMA Placement, Full Tongue Edema	1 attempt = 28 (93.3%) 2+ attempts = 2 (6.7%)	1 attempt = 30 (93.8%) 2+ attempts = 2 (6.2%)	1	[0.06, 13.70]

## Data Availability

The data sets presented in this study are available upon request from the corresponding author due to privacy and legal reasons.

## Abbreviations

The following abbreviations are used in this manuscript:

CRNAs	Certified Registered Nurse Anesthetists
Aas	Anesthesiology Assistants
DAA	Difficult Airway Algorithm
DL	Direct Laryngoscopy
LMA	Laryngeal Mask Airway
VL	Video Laryngoscopy
ROM	Range of Motion
